# Draft Genome Sequence of a “*Candidatus* Liberibacter europaeus” Strain Assembled from Broom Psyllids (Arytainilla spartiophila) from New Zealand

**DOI:** 10.1128/genomeA.00430-18

**Published:** 2018-05-17

**Authors:** Rebekah A. Frampton, Sarah M. Thompson, Falk Kalamorz, Charles David, Shea M. Addison, Grant R. Smith

**Affiliations:** a The New Zealand Institute for Plant and Food Research Limited, Lincoln, New Zealand; b Plant Biosecurity Collaborative Research Centre, Canberra, Australia

## Abstract

Here, we report the draft genome sequence of “*Candidatus* Liberibacter europaeus” ASNZ1, assembled from broom psyllids (Arytainilla spartiophila) from New Zealand. The assembly comprises 15 contigs, with a total length of 1.33 Mb and a G+C content of 33.5%.

## GENOME ANNOUNCEMENT

In 1993, the broom psyllid (Arytainilla spartiophila) was released in New Zealand as a biocontrol agent against Scotch broom (Cytisus scoparius) (1). At the end of 2011, Scotch broom plants with symptoms that included leaf curling, leaf dwarfing, and, in some cases, leaf tip chlorosis, were observed in Canterbury, New Zealand. “*Candidatus* Liberibacter europaeus” was found in the affected plants and in broom psyllids associated with the plants (2). “*Ca.* Liberibacter europaeus” was first described as an endophyte of pear trees in Italy due to the bacterium reaching high titers without causing disease (3). It is transmitted between plants by the pear psyllid (Cacopsylla pyri), in which it can also reach high titers (3). A comparison of the 16S rRNA and the 16S-23S intergenic spacer showed the bacteria from pear trees and Scotch broom to be 99.7% similar (2). In order to further investigate “*Ca.* Liberibacter europaeus” and its different hosts and lifestyles, a draft genome sequence was assembled.

“*Ca.* Liberibacter europaeus” has not, to date, been isolated and grown in axenic culture; therefore, we used an ultracentrifugation-based method to enrich for bacterial cells. Broom psyllids were collected in December 2012 from Lincoln, Canterbury, New Zealand. Five psyllids were crushed and added to a column of 30% OptiPrep medium (Sigma-Aldrich), which was then ultracentrifuged at 100,000 × *g* for 2 h. DNA extracted from each 200-µl fraction was screened with a quantitative PCR (qPCR) assay for the presence of “*Ca*. Liberibacter europaeus” using primers targeting a region of the 16S rRNA gene. The DNA from the fraction with the highest copy number was whole-genome amplified using a REPLI-g kit (Qiagen) per the manufacturer’s recommendations. This DNA was sequenced using Illumina 2000 (160 to 180-bp paired end insert; Macrogen, Inc., South Korea) and PacBio RS II (Expression Analysis, USA) technology. The Illumina sequences were assembled using SOAPdenovo2 (4). An iterative mapping approach and PacBio long reads were used to increase the length of the contigs. PCR amplification and the PacBio long reads were used to confirm the gene synteny in regions of low Illumina coverage. Illumina sequences from a broom psyllid not hosting “*Ca.* Liberibacter europaeus” were mapped to the draft genome sequence to confirm that the contigs are part of the “*Ca.* Liberibacter europaeus” genome. The resulting draft genome sequence consists of 15 contigs with a G+C content of 33.5%. The total length of the assembly is 1,331,154 bp. The low G+C content and small size of the genome are consistent with those of sequenced genomes from other Liberibacter species (5). The genome was annotated using the NCBI Prokaryotic Genome Annotation Pipeline (6). The “*Ca.* Liberibacter europaeus” ASNZ1 genome is predicted to have 1,123 coding sequences, 3 rRNA operons, and 44 tRNAs. This genome sequence will enable genomic comparisons of “*Ca.* Liberibacter europaeus” with other Liberibacter species in order to better understand the lifestyles of this emerging group of plant pathogens and endophytes.

### Accession number(s).

The draft genome sequence of “*Ca.* Liberibacter europaeus” ASNZ1 has been deposited in GenBank under accession number PSQJ00000000. The version described in this paper is the first version, PSQJ01000000.
